# The Critical Role of Bach2 in Shaping the Balance between CD4^+^ T Cell Subsets in Immune-Mediated Diseases

**DOI:** 10.1155/2019/2609737

**Published:** 2019-12-30

**Authors:** Lingyi Yang, Shuli Chen, Qiuyu Zhao, Ying Sun, Hong Nie

**Affiliations:** Shanghai Institute of Immunology, Department of Immunology and Microbiology, Shanghai Jiao Tong University School of Medicine, Shanghai, China

## Abstract

The transcription factor Bach2 which is predominantly expressed in B and T lymphocytes represses the expression of genes by forming heterodimers with small Maf and Batf proteins and binding to the corresponding sequence on the DNA. In this way, Bach2 serves as a highly conserved repressor which controls the terminal differentiation and maturation of both B and T lymphocytes. It is required for class switch recombination (CSR) and somatic hypermutation (SHM) of immunoglobulin genes in activated B cells, and its function in B cell differentiation has been well-described. Furthermore, emerging data show that Bach2 regulates transcriptional activity in T cells at super enhancers or regions of high transcriptional activity, thus stabilizing immunoregulatory capacity and maintaining T cell homeostasis. Bach2 is also critical for the formation and function of CD4^+^ T cell lineages (Th1, Th2, Th9, Th17, T follicular helper (Tfh), and regulatory T (Treg) cells). Genetic variations within Bach2 locus are associated with numerous immune-mediated diseases including multiple sclerosis (MS), rheumatoid arthritis (RA), chronic pancreatitis (CP), type 2 chronic airway inflammation, inflammatory bowel disease (IBD), and type 1 diabetes. Here, we reveal a critical role of Bach2 in regulating T cell biology and the correlation with these immune-mediated diseases.

## 1. Introduction

Transcription factors play key roles in the generation of CD4^+^ T cell diversity, and some positive regulators act to stabilize lineage commitment together with the negative regulators [1]. The BTB and CNC homolog 2 (Bach2) is one of these transcription factors that regulate transcriptional activity in T cells at super enhancers or regions of high transcriptional activity [2]. Early studies have showed its important regulatory role in B cell development and tumor immunosuppression. Recent studies have indicated that Bach2 also expresses in T cells and regulates T lymphocyte proliferation, differentiation, and immune homeostasis. Gene polymorphisms of the single gene locus encoding Bach2 are also correlated with a variety of autoimmune and allergic diseases. Motivated by these developments, we summarized the role of Bach2 in the differentiation, homeostasis, and function of CD4^+^ T cell subsets as well as the relationship between Bach2 expression and some immune-mediated diseases.

## 2. Structure and Function of Bach2

Bach2 is a transcription factor of the Bach family which gene is located on the human chromosome 6 (6q15) and mouse chromosome 4 (4A4). The Bach2-encoded protein contains 741 amino acids and its functional domains are highly conserved. The C-terminus of the Bach2 gene has a basic leucine zipper (bZip) structure, which characteristically binds to MafK, a member of Maf family proteins [2]. Therefore, the formed heterodimer provides a fitted structure to bind to the DNA consensus sequence T-MARE (TGCTGA(G/C)TCAGCA) containing the TPA response element (TRE) [2]. Upon heterodimer binding to MARE, it generally represses the expression of nearby target genes involved in the cellular transcriptional regulation process [3]. Moreover, Bach2 binds to the basic leucine zipper transcription factor ATF-like (Batf) family, which belongs to the activated protein 1 (AP-1) family, thus suggesting that Bach2 affects AP-1-mediated gene regulation. And the heterodimer formed by Bach2 and Batf is functionally related to IL-4 expression and Th2 function [4]. The Zip domain contains a nuclear localization signal that, in conjunction with the C-terminal nuclear output signal, regulates the intracellular localization of Bach2 [2]. During the oxidative stress process, cytoplasmic localization signals induce the accumulation of Bach2 in the nucleus, leading to apoptosis [5]. In B cells, heme can bind to Bach2 to inhibit its DNA binding activity and induce its degradation, thus regulating plasma cell differentiation and modulating humoral immunity [6]. SUMO-specific protease 3 (SENP3) prevents the nuclear export of Bach2 by catalyzing its deSUMOylation, repressing the genes associated with CD4^+^ T effector cell differentiation and stabilizing Treg cell-specific gene signatures [7]. At the N-terminus, Bach2 possesses a BTB/POZ domain which mediates the interaction between proteins containing this domain (homologous dimerization or heterodimerization) [3, 8].

The BTB and CNC homology (Bach) family consists of Bach1 and Bach2. Bach1 is widely expressed in various cells, especially in hematopoietic cells. Bach2 is currently only found in B cells, T cells, alveolar macrophages, and neural cells. Among them, Bach2 is highly expressed in B cells and the regulatory function in B cells has been extensively studied. It suppresses the differentiation of B cells into plasma cells by inhibiting B lymphocyte-induced maturation protein 1 (Blimp-1), which is encoded by the PRDM1 gene, thereby extending the time of somatic hypermutation and class switch. After completion of these two sections, Bach2 expression is decreased and B cells finally differentiate into plasma cells [9].

In recent years, evidences have showed that Bach2 is expressed in T cells and represses a set of genes for the effector T cell function, thereby inhibiting the differentiation of effector-memory T cells and therefore maintaining the homeostasis of T cell subsets [1, 10, 11]. All these functions are based on the structure of super enhancers (SEs). SEs are regions which possess an enhanced transcriptional activity and are predetermined to act on the establishment of the functional identity of T cell subsets. During the activation of peripheral T cells, SE regions are reported to associate with the regulation of cytokine responses. The locus encoding Bach2 emerges as the most prominent T cell SE, suggesting that Bach2 represses SE-associated genes which are critical for T cell biology [12]. In addition, Bach2 is also essential for the function of alveolar macrophages and the maintenance of pulmonary homeostasis and its gene deletion will result in alveolar protein deposition [13].

## 3. The Regulation of Bach2 on CD4^+^ T Cell Subsets

CD4^+^ T cells are fundamental in promoting or inhibiting immune-mediated pathology. After being stimulated by antigen recognition, naive CD4^+^ T cells can differentiate into distinct subsets under different conditions and perform various functions. Effector T cells are usually associated with immune-mediated diseases and are mainly divided into several distinct groups: Th1, Th2, Th9, Th17, and Tfh subsets. All of them secrete cytokines, respectively, to help regulate immune responses, especially in adaptive immunity. Th1 cells enhance phagocytic-mediated anti-infective immunity and inhibit Th2 cell proliferation while Th2 subsets limit Th1 cell proliferation. Th9 cells act in parasite infections, allergic diseases, autoimmunity, and tumor suppression mainly through secreting IL-9. Th17 cells are involved in inflammation and act especially in autoimmune diseases. Tfh cells are the subset providing B cell help during the germinal center reaction. They are the prerequisite for the generation of memory B cells and high-affinity plasma cells. Foxp3^+^ regulatory T (Treg) cells play a major role in limiting inflammatory responses and maintaining immune homeostasis by secreting cytokines or restricting target cell activation through direct contact. Effector cells are often associated with immune-mediated diseases; however, CD4^+^Foxp3^+^ Treg cells suppress immune reactions. After the analysis of mouse T cell maps, the results showed that cytokines and cytokine receptors were the dominant class of genes exhibiting SE architecture in T cells [12]. As a transcription repressor at SE regions, Bach2 can profoundly regulate the development and function of CD4^+^ T cells.

### 3.1. The Effect of Bach2 on Th1 Cells

Interleukin- (IL-) 12 was discovered in the early 1990s to play a major role for the generation of Th1 cells while the main effector cytokines of Th1 cells are IFN-*γ* and IL-2 [14]. T-bet, which is the master transcription factor of Th1 cell differentiation, promotes IFN-*γ* production through direct transcriptional activation of the IFNG gene.

The role of Bach2 in regulation of Th1 subsets is controversial. On the one hand, recent data established that the transcriptional repressor Blimp-1 can directly suppress the expression of genes such as IFNG (IFN-*γ*), TBX21 (T-bet), and BCL-6 (Bcl-6), thereby inhibiting the production of IFN-*γ*, weakening the differentiation of Th1 cells while promoting the differentiation of Th0 cells into Th2 cells [15]. In studies on B cells, it has been found that Bach2 plays an inhibitory role against Blimp-1 by binding to the MARE sequence of the PRDM1 gene [16]. Therefore, Bach2 may contribute to the differentiation of CD4^+^ T cells into the Th1 phenotype through inhibition on Blimp-1 [2]. On the other hand, Kim et al. noted that Bach2^−/−^ CD4^+^ T cells highly expressed IFN-*γ*, which was consistent with the observed elevated levels of T-bet. It was also found that expression of Th1-related genes TBX21, IFNG, and IL12B2 was upregulated in Bach2^−/−^ Treg cells [11]. Roychoudhuri et al. found that Bach2 inhibited some genes involved in effector lineage-specific functions, such as IL12RB1 (IL-12R*β*1), IL12RB2, MAP3K8, and GADD45G which are essential for Th1 cell differentiation [1]. These findings implicate the inhibitory effect of Bach2 on Th1 cells. Thus, the regulatory function of Bach2 on Th1 differentiation may be achieved through a complex regulatory network.

### 3.2. The Effect of Bach2 on Th2 Cells

Th2 cells are a subset of CD4^+^ T cells, and Gata3 is the specific transcription factor. Th2 cells secrete cytokines such as IL-4, IL-5, and IL-13 to promote the activation of eosinophils, basophils, and B cells. In this way, they play an important role in the immune response of parasitic diseases. Bach2 is deemed for a negative impact on Th2 cells. Tsukumo et al. found that even in the presence of cytokines that promote Th1 cell differentiation, there is a markedly enhanced tendency for Bach2^−/−^ CD4^+^ T cells to exhibit stronger ability to differentiate into Th2 cells and enhance expression of Th2-associated cytokines. One possible conjecture is that Bach2 can inhibit multiple genes involved in Th2 cell differentiation and function [10]. Therefore, as mentioned above, Bach2 shows a stronger negative power in Th2 cell differentiation.

The Batf family belongs to the AP-1 family and participates in the regulation of helper T cell subset differentiation. Among the Batf family, Batf3 regulates Th2 function [17]. Bach2 binds to Batf during the differentiation of naive T cells and restrains excessive differentiation of Th2 cells by two pathways. On the one hand, the Bach2-Batf complex binds to the AP-1 motif in the regulatory region of the Th2 cytokine locus and inhibits the production of Th2 cytokines. On the other hand, the Bach-Batf complex directly binds to the Batf and Batf3 loci to inhibit their transcription. A mechanism theory has been clearly expounded that in Bach2-deficient naive CD4^+^ T cells, the native highly expressed IL-4 induces the combination of Batf and interferon regulatory factor 4 (Irf4), which in turn enhances IL-4 expression. IL-4 and Batf-Irf4 complex forms a positive feedback amplification loop that induces Th2 differentiation [4]. Moreover, spontaneous lung-specific Th2-type allergic inflammation in T cell-specific Bach2^−/−^ mice suggests Bach2 may restrain Th2 inflammation. Compared with wild-type mice in the control group, the number of T cells producing IL-5 and IL-13 increased in the lungs of Bach2^−/−^ mice [4]. In addition, in the lung cells of T cell-specific Bach2^−/−^ mice, the number of IL-33R*α*^+^CD4^+^ T cells which are pathogenic Th2 with high Th2 cytokines expression increased. These results suggest that Bach2 is involved in Th2-mediated chronic inflammation [18].

### 3.3. The Effect of Bach2 on Th9 Cells

Th9 cells are a recently discovered subset of CD4^+^ T cells, defined by their high secretion of IL-9. IL-9 and IL-9-producing cells play a role in helminth infections, allergic diseases (such as asthma), autoimmunity, and tumor suppression. The differentiation of Th9 cells from naive T cells is dependent on IL-4 and TGF-*β*. The development of Th9 cells also requires a network of transcription factors including Stat6, Irf4, Batf, PU.1, and Stat5.

Although Bach2 represses the production of multiple cytokines such as IL-4, IL-13, and IFN-*γ*, it has been observed that Bach2 promotes IL-9 production in Th9 cells. This positive regulation is achieved by directly binding to the Il9 gene and enhancing IL-9-inducing transcription factor (including Batf, Irf4, Junb, and Jun) expression in Th9 cells [19]. The knockdown of Bach2 significantly decreases IL-9 production and other IL-9-inducing transcription factors. Contrary to the inhibitory effect of the Bach2-Batf complex on Th2 cytokines, Bach2 facilitates the ability of Batf to promote IL-9 production in Th9 cells [19]. Additionally, Bach2 transduction promotes IL-10 production in Th9 cells, despite the repressive regulation of Bach2 on IL-10 in naive CD4^+^ T cells and induced Tregs [10, 11, 19]. But Bach2 knockdown has no effect on Il10 expression in Th9 cells [19]. Therefore, different from suppressing cytokine secretion in effector T cells, Bach2 increases IL-9 and IL-10 production in Th9 cells.

### 3.4. The Effect of Bach2 on Th17 Cells

Th17 cells secrete cytokines such as IL-17, IL-22, and IL-23, recruit neutrophils, promote inflammation, and clear extracellular pathogens. Therefore, this cell subset plays a critical role in the occurrence of various autoimmune diseases. ROR*γ*t is its iconic transcription factor. In Bach2^−/−^ mice, CD4^+^ T cells differentiated into Th1, Th2, and Th17 cell subsets preferentially even under conditions conducive to iTreg [1]. In the case of chronic pancreatitis (CP), Rorc (ROR*γ*t) was higher in CD4^+^ T cells isolated from pancreatic tissues, indicating that the naive T cells of CP patients were more likely to differentiate into Th17 cells. Inversely, the expression of Bach2 was lower. Sasikala et al. believed that the reduction in Bach2 expression in CP caused CD4^+^ T cells to differentiate into Th17 cells [20].

The initial state maintenance of Th17 cells and cellular inflammation adjustment are part of the effects of Bach2. Hoppmann et al. noted that Bach2 expression was higher in naive T cells while decreased in normal Th17 cells and Th17 cells of EAE mice (Th17eae), implying that Bach2 can maintain the initial state by inhibiting effector memory-related genes [21]. They suggested that the calcium-binding protein S100A may be a direct target of Bach2 and found that S100A levels were elevated in Bach2^−/−^ mice. The experiment showed that the levels of S100A in the naive T cells, normal Th17, and Th17eae cells increased sequentially, which was negatively correlated with Bach2 expression. S100A strengthens the severity of inflammatory or autoimmune diseases such as atherosclerosis and rheumatoid arthritis (RA). In brief, the observed downregulation of Bach2 and increased levels of S100A may be associated features of autoimmune neuroinflammation [21].

Many single-nucleotide polymorphisms (SNPs) were found on the Bach2 exon sequence. Among them, two SNPs (rs9111 and rs45553631) on exons participate in Bach2 inhibition. In the CP case study, the exposure of different genotypes of T cells to CP tissue extracts demonstrated that the degree of Bach2 inhibition was affected by both genotype and inflammatory environment. And under this circumstance, Bach2 inhibition may contribute to the differentiation of CD4^+^ T cells into pathogenic Th17 cells, resulting in the development of CP [20]. Other studies have confirmed the association between some intronic SNPs and immune-mediated diseases including celiac disease, type 1 diabetes, RA, and autoimmune Addison's disease [20, 22].

### 3.5. The Effect of Bach2 on Tfh Cells

T follicular helper (Tfh) cells are a specialized CD4^+^ T cell subset, which is mainly present in peripheral lymphoid follicles. They play a crucial role in the development of immunity by controlling the germinal center (GC) formation and the cellular reactions occur in GC. And they are key cells for GC B cell responses, especially in the differentiation of B cells into plasma cells, antibody production, and Ig class switch. Tfh cells are characterized by high expression of the lineage-defining transcription factor Bcl-6, the chemokine receptor CXCR5, the coinhibitory receptor PD-1, and interleukin-21 (IL-21) [23, 24].

Bach2 is downregulated and low expressed in Tfh cells [25, 26]. Bach2 is a crucial negative regulator of Tfh cells to remain its steady state and restrain its pathogenic accumulation [25]. It critically regulates Tfh cell differentiation. The deletion of Bach2 results in preferential Tfh cell differentiation even enhanced non-Tfh cell effector functions [27]. And Bach2-deficient Tfh cells were altered to the IL-4-producing subset, which induced IgG1 and IgE isotype switching of B cells [26]. Overexpression of Bach2 resulted in a fast loss of Tfh cell phenotype and subsequent breakdown of the GC response [25]. In GC B cells, Bach2 is known to directly repress the transcription factor Blimp-1 and negatively regulates its expression, while Blimp-1 antagonizes Bcl-6 [28, 29]. As a result, high Bach2 expression levels in GC B cells prevent Blimp-1 expression and ensure Bcl-6 expression [9]. Bcl-6 and Bach2 are crucial for GC B-cell fate and are known to interact and repress transcription of PRDM1, a key driver of plasma cell differentiation [30]. Contrary to the GC B cells, Bach2 in Tfh cells does not regulate Bcl-6 indirectly via repression of Blimp-1. Instead, Bach2 is downregulated to prevent direct inhibition of Bcl-6 by binding to its promotor region by displacing an activating complex of Irf-4 and Batf [25]. And Bach2 is directly suppressed by Bcl-6 in turn.

Bach2 can also directly downregulate the transcription of CXCR5 and c-Maf [26]. The deletion of Bach2 leads to the induction of CXCR5 expression even before the upregulation of Ascl2, which has been reported to initiate the Tfh cell differentiation programming and directly regulate CXCR5 expression [27, 31]. There is a regulatory element 36 kb upstream of the murine CXCR5 locus that suppresses the CXCR5 promoter activity in a Bach2-dependent manner. The downregulation of Bach2 may be necessary yet not enough for the CXCR5 expression. CXCR5 overexpression cannot rescue Bach2-mediated suppression of Tfh cell differentiation [27]. But Bach2 does not regulate Tfh cell proliferation, survival, or migration [25]. In conclusion, Bach2 is a negative regulator for Tfh cells and suppresses the promoters of CXCR5 to downregulate its expression.

### 3.6. The Effect of Bach2 on Regulatory T Cells

Regulatory T (Treg) cell is a kind of T cell subset with immunosuppressive function, which can secrete anti-inflammatory cytokines such as IL-10 and TGF-*β* and control the activity of various immune cells, thus inhibiting immune responses and preventing autoimmune diseases [32]. The two major subpopulations of Treg cells are naturally regulatory T (nTreg) cells and induced regulatory T (iTreg) cells. The former cell subsets develop from the naive T cells in the thymus, whose phenotype is CD4^+^CD25^+^Foxp3^+^ Treg. The latter ones develop after the stimulation of T cells in peripheral tissues by TGF-*β* and IL-2. At present, the highly expressed transcription factor Foxp3 is considered as a marker of Treg cells, but it has also been observed lowly expressed in other activated T cells in humans [33].

Bach2 regulates the development and differentiation of Treg cells and is required for the efficient production of nTreg and iTreg cells. Treg cell formation has a dose-dependent relationship with Bach2. A study found that compared to Bach2 homozygous mice, Treg cell frequency decreased in heterozygous ones. It was also found that the number of Treg cells in Bach2^−/−^ mice decreased significantly, which eventually lead to a series of diseases [1]. Moreover, Bach2 regulates peripheral iTreg development. By tracking the naive CD4^+^ T cells transferred into the rag1^−/−^ host, Roychoudhuri et al. observed that compared to wild-type mice, the number of CD4^+^ T cells converted to Foxp3^+^iTreg was obviously reduced in Bach2^−/−^ mice. In vitro, the naive CD4^+^ T cells of Bach2^−/−^ mice were notably impaired in their ability to form Foxp3^+^iTreg under TGF-*β* stimulation. Recombinant expression of Bach2 via retroviral transduction can ameliorate the induction defect of iTreg in Bach2^−/−^ mice, which also confirms that the iTreg cell formation requires the participation of Bach2 [1]. Studies have showed that Bach2-inhibited transcription factor Blimp-1 can drive the complete effector differentiation of CD4^+^ T cells and limit their differentiation into Treg cells [28]. In addition to that, many effector-related genes inhibited by Bach2 can encode signaling proteins that antagonize Treg cell differentiation, including IL12RB1, IL12RB2, and TNFSF4 [32, 34]. Through these inhibitory effects, Bach2 stabilizes Treg cell development and promotes Treg cell differentiation to suppress immune activation and maintain immune homeostasis [1].

Moreover, Bach2 plays an indispensable role in the homeostasis maintenance and function of Treg cells. Bach2 can enhance the adaptability of thymus and peripheral Foxp3^+^ Treg cells and improve their survival. Bach2^−/−^ Treg showed an activated phenotype, with elevated expression levels of CD44 and GITR [11]. Vinuesa et al. found that the ratio of Bim to Bcl-2 was prominently raised in Bach2^−/−^ Treg cells, indicating compromised cell survivability [23]. And the mRNA level of antiapoptotic molecule Mcl-1 was decreased, with most cells showing the apoptotic Annexin V^+^ phenotype [35, 36], while the expression of the proliferation marker Ki67 was notably higher, which may be the compensatory proliferation under destroyed Treg cell maintenance condition [11]. Therefore, Bach2 maintains Treg cells' homeostasis by regulating their activation, proliferation, and apoptosis.

Foxp3 is a crucial transcription factor of Treg cells. Treg cell development and function depend on the stable expression of Foxp3 [37, 38]. Animals reconstituted with Bach2^−/−^ bone marrow (BM) emerged mucosal thickening of the large intestine with infiltration of Bach2^−/−^ cells, profound weight-loss, and diminished survival, while cotransfer of WT BM prevented these pathological changes. The BM from mice, which bears the complete Bach2 gene but lacks the functional Foxp3 protein, still caused the pathological phenotype. Consequently, Treg cells' immunomodulatory effects depend on the presence of Foxp3 [1]. Furthermore, Foxp3 expression level in Foxp3^+^ Treg cells of Bach2^−/−^ mice was significantly lower [11]. This might be related to higher expression of TNFSF4 and TNFRSF4 (encodes OX40L and OX40, respectively), a receptor/ligand pair which inhibits Foxp3 expression [39]. Therefore, Bach2 is required for the complete expression of Foxp3 in Treg cells, but the specific intracellular mechanism is still unclear [11]. It is also speculated that Bach2 may be a target gene of Foxp3 [11, 40]. Most researchers suggest that Bach2 regulates Treg cells' homeostasis and function by modulating the expression of various transcription factors such as Foxp3, Tcf1, and Blimp-1.

## 4. The Connection between Bach2 and Immune-Mediated Diseases

Immune-mediated diseases, such as RA, multiple sclerosis (MS), and systemic lupus erythematosus (SLE), which are influenced by both genetic susceptibility and environmental modulation, are characterized by the loss of self-tolerance, resulting in immune-mediated tissue destruction. Inflammation is another characteristic of these diseases, which involves a group of cytokines, chemokines, and interactions between CD4^+^ T helper cells (e.g., Th1, Th2, Th9, Th17, and Tfh) and regulatory T cells (Tregs). Gene polymorphisms of the single gene locus encoding Bach2 are also correlated with a variety of immune-mediated diseases including multiple sclerosis (MS) [41], RA [42], CP [20], type 2 chronic airway inflammation [18], inflammatory bowel disease (IBD) [43], and type 1 diabetes [44]. Significantly, both proinflammatory and anti-inflammatory molecular mechanisms are involved in the variant expression of Bach2 and these diseases.

### 4.1. Multiple Sclerosis

MS is a complex central nervous system (CNS) disease characterized by autoimmune demyelination and neurodegeneration under inflammatory action [45]. MS is the result of a joint action of genes and multiple factors. To date, genome-wide association studies have identified hundreds of risk sites for autoimmune diseases, confirming that HLA is the major susceptible gene for MS, while other genes such as BACH2, PTGER4, RGS1, and ZFP36 also exhibit strong susceptible correlations [41]. Analysis of the genome-wide function of Bach2 reveals that it can inhibit genes associated with T cell proliferation [46]. Furthermore, Bach2 is directly involved in the upregulation of Foxp3 gene expression and its function enhancement [11]. Treg-dependent inflammatory inhibition is required in the EAE mouse model, where the Bach2 gene expression was significantly downregulated [46]. Similarly, Bach2 transcription levels in whole blood of MS patients are reduced compared to healthy controls [33]. It has been proven that the immunomodulatory function of Bach2 is impaired in MS [47]. As a key regulator of CD4^+^ T cell differentiation, Bach2 may prevent inflammatory diseases such as MS by controlling immune tolerance and regulating the balance between immune cells [1].

### 4.2. Rheumatoid Arthritis

RA is a chronic autoimmune disease that causes progressive joint destruction and comorbidities in blood vessels, bones, and metabolism. It is characterized by chronic inflammation and associated tissue remodeling and damage. Adaptive immune cells, especially CD4^+^ memory T cells, as well as B cells and autoantibodies, play a leading role in this disease [48]. Genetic studies of RA in European ancestry populations, including WGAS and meta-analysis-based works, have identified up to 46 RA risk loci including Bach2 [49]. The type 1 diabetes-associated loci Bach2/rs11755527 were observed to be relevant to RA in Pakistani patients but with no significant relevance in British Caucasian [50, 51]. In addition, only one SNP, rs72928038 of the Bach2 intron, reached a genome-wide significance in the meta-analysis [42]. In summary, Bach2 polymorphisms are important contributors to susceptibility to the disease.

### 4.3. Chronic Pancreatitis

CP is a long-term pancreatic inflammation that alters the shape of the pancreas and affects its function, causing many pathological reactions such as malabsorption. It can be transformed from acute pancreatitis, though the manner and process of transformation are unclear [52]. Studies showed that Th1 and Th17 cells are elevated in peripheral and pancreatic tissues of patients with pancreatitis [53, 54]. Sasikala et al.'s study of the relationship between CP and Bach2 showed that the decrease in the expression of Bach2 in circulation and pancreatic tissues of patients with CP was associated with an increase in the differentiation of Th17 and an abnormal increase in the number of Th17 cells involved in the induction of inflammatory responses [20]. Another result of the study showed that the allelic frequency of an intron single-nucleotide polymorphism (SNP) and an exon SNP of Bach2 differed in the risk of CP and pancreatogenic diabetes, respectively [20]. It is suggested that as a susceptibility gene for many diseases, Bach2 is worthy of further exploration of the relationship between the SNPs and their related diseases.

### 4.4. Type 2 Chronic Airway Inflammation

The onset of type 2 chronic airway inflammation is closely related to the dysregulation of Th2 cells. Bach2 is important in regulating Th2 cell differentiation and Th2 cell-mediated type 2 immune responses [10]. Repetitive antigen and cytokine stimulation reduces the Bach2 level via the constitutive activation of the PI3K-Akt-mTOR signaling, leading CD4^+^ T cells to induce antigen-independent Th2 cytokine production and subsequent type 2 inflammation [18]. First, Bach2 associates with Batf and binds to the Th2 cytokine gene locus, thereby inhibiting Th2 cytokine production and Th2 cell differentiation. The deletion of Batf ameliorated the spontaneous development of type 2 airway inflammation that is found in mice with Bach2 deficiency specifically in T cells [4]. Second, Bach2 inhibits IL-33-dependent Th2 cytokine production through suppressing Il1rl1 transcription and the generation of IL-33R*α*^+^CD4^+^ T cells [18]. Taken together, there is a critical role for Bach2 in regulating Th2 cell differentiation and the subsequent onset of type 2 chronic airway inflammation.

### 4.5. Inflammatory Bowel Disease

Inflammatory bowel disease (IBD) is a chronic intestinal inflammatory condition, with two main clinical phenotypes: Crohn's disease (CD) and ulcerative colitis (UC). Genome-wide association studies (GWAS) indicate that Bach2 is a susceptibility gene of IBD, with the risk locus rs1847472 in both CD and UC [43, 55]. The role of Bach2 in IBD may be associated with its effect in inducing immunoglobulin class switching, thus affecting B cell IgA production, which is a central pathway in mucosal immunity [56]. Laffin et al. studied a cohort of patients with CD who underwent intestinal resection and identified Bach2 as a susceptibility locus for postoperative recurrence of CD. They hypothesized that a Bach2 mutation can make the patient at increased risk of an inappropriate inflammatory response to microorganisms after intestinal resection [57]. Although variation in Bach2 shows more relevant to CD, Christodoulou et al. examined two patients with severe UC and observed they both carried unique, potentially detrimental mutation in Bach2 gene [58]. Therefore, Bach2 may be a potential candidate for therapy of inflammatory bowel diseases.

### 4.6. Type 1 Diabetes

Type 1 diabetes is a chronic autoimmune disease that causes insulin deficiency due to immune system-mediated destruction of pancreatic *β*-cells. CD4^+^ and CD8 ^+^ T cells play an important role in the pathogenesis of this disease, and usually, autoantibodies against islet proteins can be detected [59]. Linkage and GWAS have identified more than 50 loci associated with the risk of type 1 diabetes in the human genome, and BACH2 is one of them [60]. Different SNPs of BACH2 which is associated with the genetic risk of type 1 diabetes have been found in multiple independent populations. For example, BACH2/rs3757247 is associated with childhood-onset type 1 diabetes and insulin-triggered type 1 diabetes in Japan [59, 61]. In addition, rs3757247 and rs11755527 in tight linkage disequilibrium among Caucasians and rs11755527 among Pakistanis are associated with type 1 diabetes [50, 62, 63]. In a multiethnic case-based experiment, genetic identification revealed that BACH2/rs11755527 was associated with protection from islet autoantibody IA-2A positivity [64].

Marroqui et al. found that there was a similar expression of Bach2 in islet cells to immune cells and Bach2 transcription factor binding sites were ubiquitous in cytokine-treated human islet mRNA [65]. In addition, Bach2 silencing and exposure to proinflammatory cytokines can increase phosphorylation of the proapoptotic protein JNK1 by upregulating mitogen-activated protein kinase 7 (MKK7) and downregulating PTPN2. JNK1 increases phosphorylation of the proapoptotic protein BIM, and both JNK1 and BIM knockdown protects *β*-cells from cytokine-induced apoptosis in Bach2-silenced cells [65, 66]. Therefore, they believe that Bach2 regulates proinflammatory cytokine-induced *β*-cell apoptosis by regulating the JNK1/BIM pathway, whereas Bach2 overexpression has a protective effect [65]. In conclusion, many experiments have confirmed that Bach2 is associated with the genetic risk of type 1 diabetes.

## 5. Conclusion

**Figure 1:**
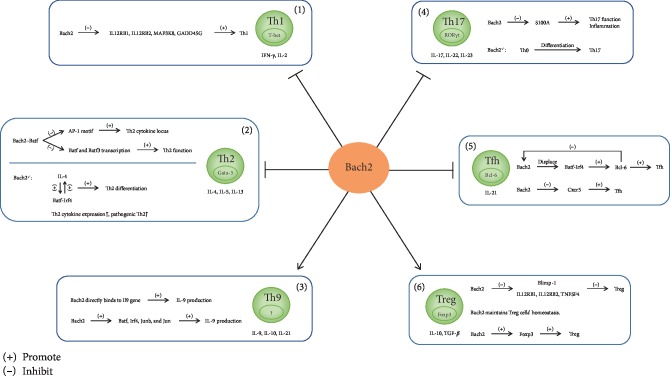
Effect of transcription factor Bach2 on CD4^+^ T cell subsets. (1) Bach2 inhibits Th1 cell differentiation. Bach2 inhibits some genes such as IL12RB1 (IL-12R*β*1), IL12RB2, MAP3K8, and GADD45G, which are essential for Th1 cell differentiation, and then inhibits the differentiation of Th1. (2) Bach2 inhibits Th2 cell differentiation and function. Bach2 binds to Batf, and the Bach2-Batf complex restrains the differentiation of Th2 by two pathways. The Bach2-Batf complex directly binds to the AP-1 motif in the regulatory region of the Th2 cytokine locus and inhibits the production of Th2 cytokines. The Bach2-Batf complex also binds to Batf and Batf3 loci to inhibit their transcription, while Batf3 regulates Th2 function. In Bach2^−/−^ naive CD4^+^ T cells, IL-4 and Batf-Irf4 complex form a positive feedback amplification loop that induces Th2 differentiation. In T cell-specific Bach2^−/−^ mice, the expression level of Th2 cytokines was elevated, and in the lung cells, the number of pathogenic Th2 cells (IL-33R*α*^+^CD4^+^ T cells) with high cytokine expression also increased. (3) Bach2 promotes Th9-associated cytokine secretion. Bach2 promotes IL-9 production in Th9 cells by directly binding to the Il9 gene and by indirectly upregulating the expression of IL-9-inducing transcription factor, such as Batf, Irf4, Junb, and Jun. (4) Bach2 inhibits Th17 differentiation. S100A is a direct target of Bach2. S100A strengthens the severity of inflammatory and Th17 differentiation. Bach2 inhibits Th17 differentiation through binding to S100A. In Bach2^−/−^ mice, the naive T cells were more likely to differentiate into Th17 cells. (5) Bach2 inhibits Tfh differentiation. Bach2 displaces the complex of Irf-4 and Batf that activates Bcl-6 promoter. Bach2 is also suppressed by Bcl-6 in turn. Bach2 directly downregulates the transcription of Cxcr5, the chemokine receptor of Tfh. (6) Bach2 promotes Treg differentiation. Bach2-inhibited transcription factor Blimp-1 can limit CD4^+^ T cell differentiation into Treg cells. In addition to that, many effector-related genes that Bach2 inhibited can encode signaling proteins that antagonize Treg cell differentiation, including IL12RB1, IL12RB2, and TNFSF4. Moreover, Bach2 plays an indispensable role in the homeostasis maintenance and function of Treg cells. And Bach2 is required for the complete expression of Foxp3, in which Treg cells' immunomodulatory effects depend on.

In this review, we have expounded that Bach2 is an important immune-related transcription factor in Th1, Th2, Th9, Th17, Tfh, and Treg subsets and has multiple function in the regulation of CD4^+^ T cells during inflammation development (Figure 1). Nevertheless, whether the change of Bach2 expression has a direct influence on the occurrence of these chronic inflammatory diseases is still worth studying. Further researches and in-depth understanding will help to explore the pathogenesis of these diseases and open new strategies for treatment.
